# Knowledge, attitude, and associated factors towards COVID-19 among nurses who work in South Gondar Zone, hospitals, Northwest Ethiopia 2020. A multi-central institution-based cross-sectional study

**DOI:** 10.1016/j.nmni.2021.100914

**Published:** 2021-07-03

**Authors:** D.G. Feleke, E.S. Chanie, B.A. Tilaye, D. Mesfin, B.M. Birhane, W.A. Bayih, S.F. Tassew, S. Asnakew, T.A. Berlie, T. Dires, E. Dagnaw, T.Y. Tadesse

**Affiliations:** 1) Department of Pediatrics and Child Health Nursing, College of Health Sciences, Debre Tabor University, P.O.BOX 272, Debre Tabor, Ethiopia; 2) Department of Maternity and Neonatal Nursing, College of Health Sciences, Debre Tabor University, P.O.BOX 272, Debre Tabor, Ethiopia; 3) Department of Emergency Medicine and Critical Care Nursing, College of Health Sciences, Debre Tabor University, P.O.BOX 272, Debre Tabor, Ethiopia; 4) Department of Psychiatry, College of Health Sciences, Debre Tabor University, P.O.BOX 272, Debre Tabor, Ethiopia; 5) Department of Adult Health Nursing, College of Health Sciences, Debre Tabor University, P.O.BOX 272, Debre Tabor, Ethiopia; 6) Department of Midwifery, College of Health Sciences, Debre Tabor University, P.O.BOX 272, Debre Tabor, Ethiopia; 7) Department of Pharmacy, College of Health Sciences, Debre Tabor University, P.O.BOX 272, Debre Tabor, Ethiopia

**Keywords:** Attitude, coronavirus-2019, Ethiopia, knowledge, nurse

## Abstract

Coronavirus disease 2019 is an emerging respiratory disease that is caused by a novel coronavirus and was first detected in December 2019 in Wuhan, China. The world was affected by the Coronavirus Disease in 2019. In sub-Saharan Africa, including Ethiopia, there is no study conducted on the level of Knowledge, Attitude, and Associated Factors towards Coronavirus disease 2019 among Health care workers, specifically Nurses. This study aims to assess the level of Knowledge, Attitude, and Associated Factors towards Coronavirus disease 2019 among Nurses who work in South Gondar Zone, Hospitals, Northwest Ethiopia, 2020. An Institution based cross-sectional study was conducted among 166 Nurses in South Gondar Zone, Ethiopia, From 1 June to 30 June 2020. For selecting the study participants after proportional allocation of study subjects to each hospital, simple random sampling techniques were to be used. Data were entered into Epi info version 7.2.0.1, and exported to Statistical Package for Social Sciences window version 24 for analysis. Binary and multivariable logistic regression was used to see the association between dependent and independent variables. Adjusted odds ratio with 95% confidence interval was computed. P-value < 0.05 was used to declare association. Finally, the result is presented in the form of texts, tables, and graphs. Of 166 Nurses, 166 (100% response rate) responded to the online interview questionnaire. Of the participating 166 nurses, 57.2% were females and 42.8% were males; 41.6 % of the respondents were between the ages of 20 and 29 years. About 84.9 % had good knowledge and 63.3% favourable attitude of COVID-19. Wearing general medical masks can prevent one from acquiring infection by the COVID-19 virus. AOR = 0.44, 95% CI = 0.005–0.362 were factors of knowledge about COVID-19, whereas, ‘I strongly agree’ Medical staff were ready to participate in anti-epidemic in the community, AOR = 0.08, 95% CI = 0.003–1.76 were factors of attitude about COVID-19. Where factors of attitude about COVID-19. In this study, most of the nurses had good knowledge and a favourable attitude regarding of COVID-19. Wearing general medical masks that can prevent one from acquiring infection by the COVID-19 virus were factors in association with the knowledge of nurses on COVID-19. Similarly, Medical staff were ready to participate in anti-epidemic community factors associated with the attitudes of nurses on COVID-19.

## Background

Coronavirus disease 2019 (COVID-19) is an emerging respiratory disease caused by a novel coronavirus and was first detected in December 2019 in Wuhan, China [[1], [2], [3]]. The novel coronavirus is very similar in symptomatology to other viral respiratory infections [1,4]. The novel COVID-19 was first reported in December 2019 as a cluster of acute respiratory illness in Wuhan, Hubei Province, China, from where it spread rapidly to over 198 countries. It was declared as a global pandemic by WHO on 12th March 2020 [5,6]. COVID-19 is a new disease that is a large family of viruses that are common in people and many species of animals, including camels, cattle, cats, and bats [7]. Nowadays, COVID-19 is a life-threatening agent spread worldwide, and it has become an international concern. Health workers, especially nurses, have close contact with infected patients and have a decisive role in infection control [8]. The newest member of the COVID-19 family has been recently identified, which results in acute and severe respiratory syndrome in humans [9]. The first infected patient who had clinical manifestations such as fever, cough, and dyspnea was reported on 12th December 2019 in Wuhan, China [9,10]. Since then, COVID-19 has spread rapidly to other countries via different ways such as air travel, and now, COVID-19 is the world's pandemic problem [11]. COVID-19 has become a great public health concern in the world. No antiviral agents have been recommended so far, and prevention is the best way to limit the infection [12,13]. It seems that the current widespread outbreak has been partly associated with a delay in diagnosis and poor infection control procedures [14].

As of 22nd August 2020, over 23,266,1431 cases of COVID-19 have been reported with a death toll of over 805,863 patients, and 15,817,397 cases are recovered in the world [15]. In studies conducted in different countries such as Iran, more than half of the nurses, 56.5%, had good knowledge of COVID-19 [16], and in another study done in Iran, the overall achieved knowledge score regarding COVID-19 characteristics was 90%, with 60.8% of the general population having moderate knowledge of the disease [17]. In the study conducted in Pakistan, HCWs have good knowledge of 93.2%, and positive attitude (8.43 ± 1.78) regarding COVID-19 [18]. In a study done in Wuhan, Hubei province, China, the overall correct rate of the knowledge questionnaire was 90% [19]. In the study done at Makerere University Teaching Hospitals, Uganda, overall, 69% had sufficient knowledge, 21% had positive attitude towards COVID-19 [20]. The study conducted at District 2 Hospital, Ho Chi Minh City, showed a mean score of knowledge and attitude of 8.17 ± 1.3 (range 4–10) and 1.86 ± 0.43 (range 1–5), respectively [21].

The outbreak of COVID-19 in Ethiopia was officially confirmed on 13 March 2020 [[22], [23], [24]]. In Ethiopia, updated as of 22 August 2020, 39,033 confirmed cases were reported, of which 14,480 patients recovered and 662 deaths occurred [23].

Knowledge and Attitude towards COVID-19 can be affected by a multitude of interrelated factors [25]. A poor understanding of the disease among HCWs can result in delayed identification and treatment leading to the rapid spread of infections. Over 100 health workers have lost their lives to COVID−19, a tragedy to the world and a barrier to fight against the disease [26]. Guidelines for HCWs and online refresher courses have been developed by WHO, CDC, and various governmental organizations in various countries to boost knowledge and prevention strategies [27].

The battle against COVID-19 is still continuing in Ethiopia. To guarantee the final success, people's adherence to these control measures is essential, which is largely affected by their knowledge, attitudes towards COVID-19 in accordance with KA theory [28,29].

**Fig. 1:**
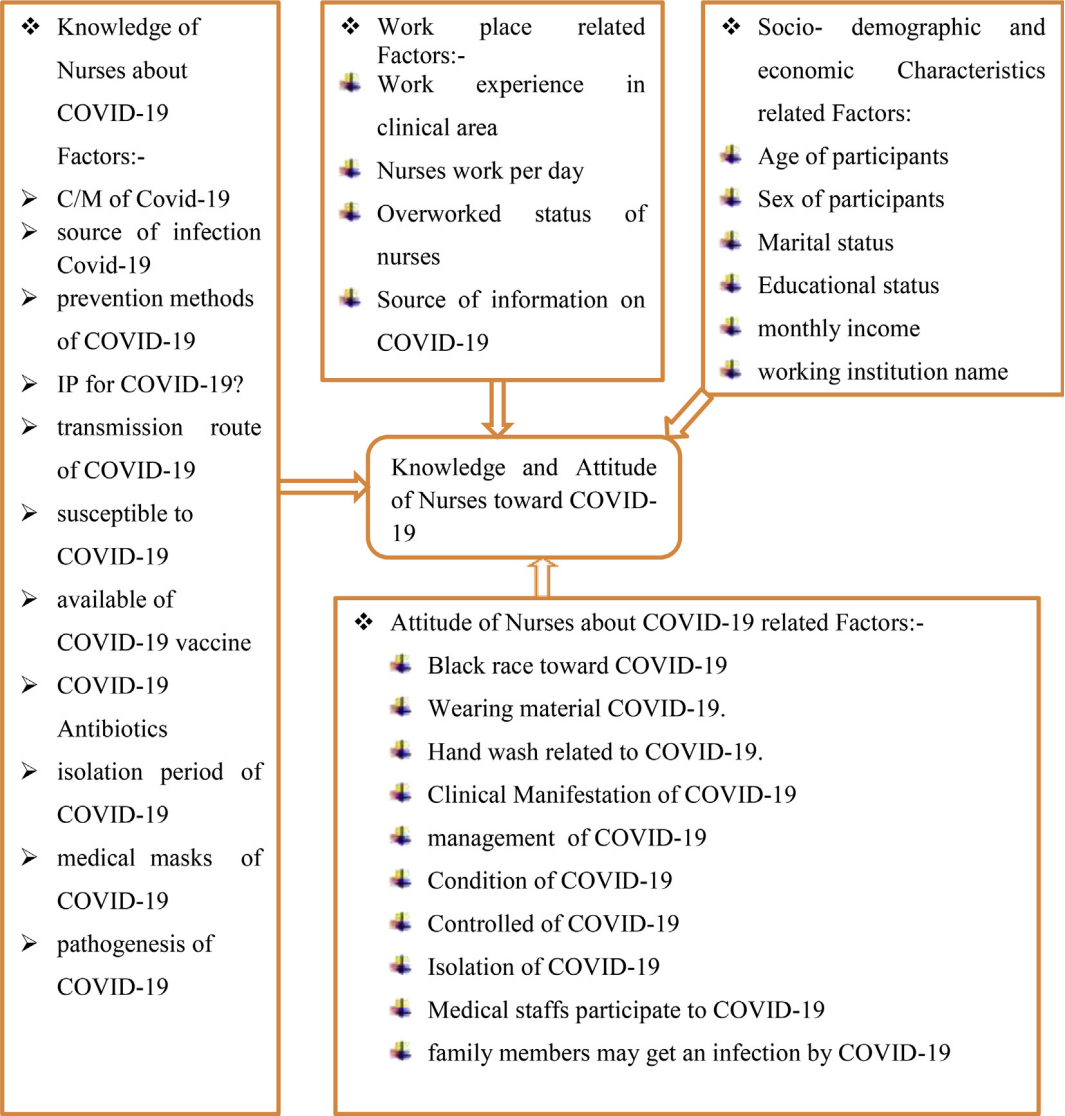
: Conceptual framework of Assessment of Knowledge, Attitude, and Associated Factors towards COVID-19 among Nurses Who Works in South Gondar Zone, Hospitals, and Northwest Ethiopia 2020. (Adapted from different articles ([16], [17], [18], [19], [20], [21], 30)).

As transmission within hospitals and protection of HCWs are important steps in the epidemic, the understanding of having enough information regarding sources, clinical manifestations, transmission routes, and prevention ways among HCWs can play roles for this gal assessment. Since nurses are in close contact with infected people, they are the main part of the infection transmission chain, and their knowledge of COVID-19 prevention and protection procedures can help prevent the transmission chain. There is a paucity of literature on the KA of HCWs towards the COVID-19 pandemic. To our knowledge, no study has been done in sub-Saharan Africa including Ethiopia to assess KA towards COVID-19, specifically among HCWs, and especially nurses to play critical roles in the prevention of COVID-19. Ethiopia is one of the most epidemic countries for COVID-19, and there is no information regarding the awareness and attitude of Ethiopian nurses about this infectious disease. Therefore, this study was aimed to assess Knowledge, Attitude, and Associated Factors towards COVID-19 among Nurses who work in South Gondar Zone, Hospitals, Northwest Ethiopia, 2020 (Sees Fig. 1 Conceptual framework).

## Methods

### Study area and period

The study was being conducted in South Gondar Zone. South Gondar Zone is one of the 11 Zones of the Amhara National Regional State and has a total of 18 woredas. Based on the information from South Gondar Zone Administrative Health Bureau, the total population in South Gondar Zone is 2,609,823, and among them 49.9% are males, and 50.1% are females. The study was conducted from 1 June to 30 June, 2020.

### Study design, and participants characteristics

An institution-based cross-sectional quantitative study was conducted. All nurses working in South Gondar Zone Hospitals were the source population of the study, and of them, selected Nurses were the study population of the study. Nurses working in South Gondar Zone Hospitals and available during the data collection period were included in the study, and Nurses who are on Annual leave and sick leave during data collection were excluded from the study.

### Sample size determination and sampling procedure

The sample size is determined using a single population proportion formula using the proportion of Health workers Level of knowledge (89%) in a study conducted in China [30] with a 95% confidence interval and precision level of 5%.

$$\text{ni}\;=\;\frac{\left(\text{Z α}/2\right)2\text{ p }\left(1-\text{P}\right)}{{\text{d}}^{2}}=\frac{\left(1.96\right)2\text{∗ }0.89\left(1-0.89\right)}{0.05^{2}}=151$$

where n = Sample size needed

z = Standard normal variable at 95% confidence level (1.96)

p = the Level of Nurses Knowledge in Iran (0.89)

d = Margin of error (0.05)

Z α/2 = Value of standard normal distribution corresponding to a significant level of alpha (α) 0.05, which is 1.96. Then add 10 % (contingency) = 166.

An overall sample size of 166 Nurses was required for the study.

**Fig. 2:**
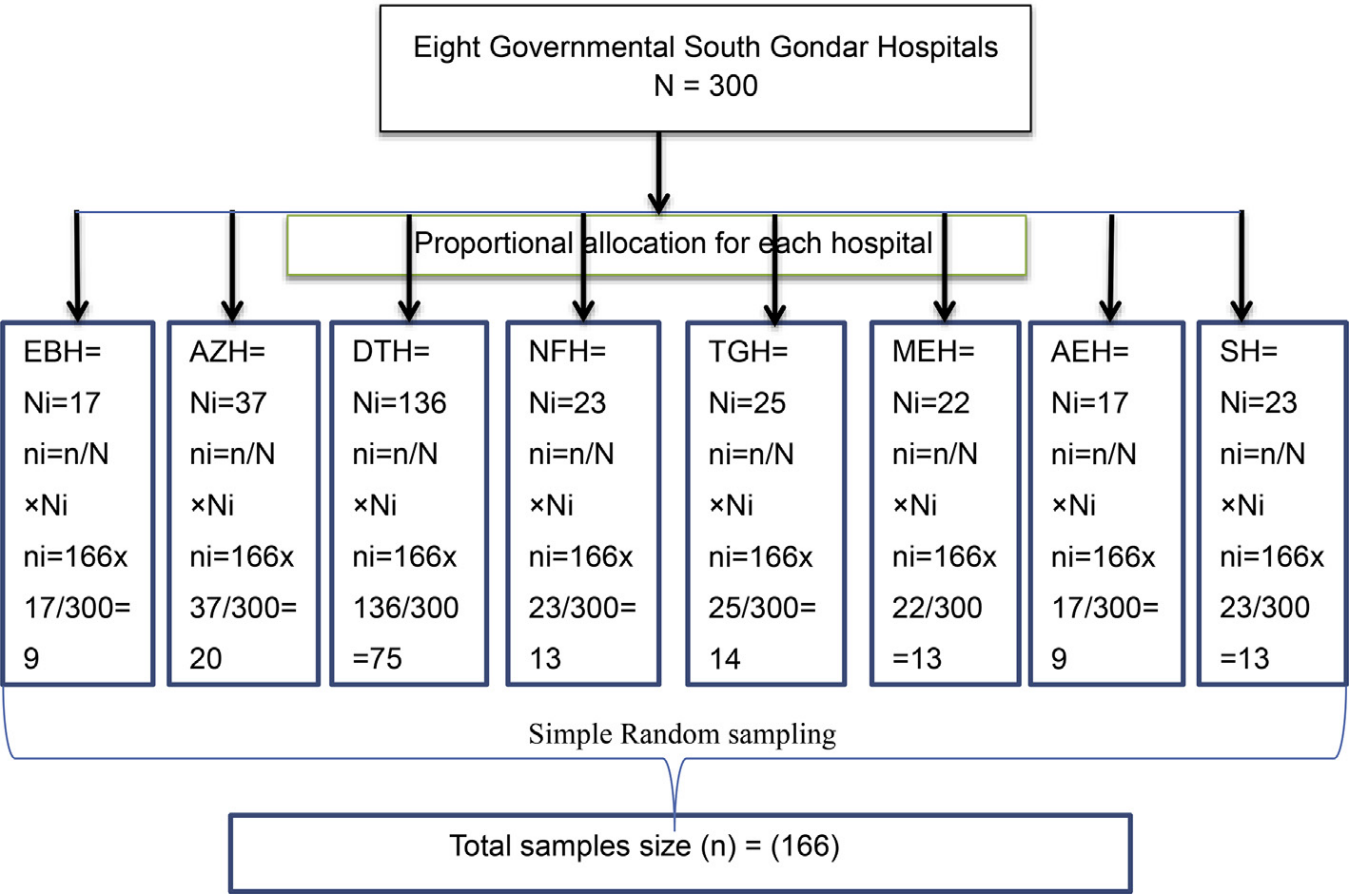
Schematic Presentation of the Sampling Procedure for Knowledge, Attitude, and Associated Factors towards COVID-19 among Nurses Who Works in South Gondar Zone, Hospitals, and Northwest Ethiopia 2020. **Key**: EBH: Ebnat Hospital, AZH: Adiss Zemen Hospital, DTH; Debre Tabor Hospital, NFH: Nifas Mewucha Hospital, TGH:Tach Gayint Hospital, MEH: Mekan Eyesus Hospital, AEH: Andabet Etie Hospital, SH: Simada Hospital, N: Total number of Nurses in the South Gondar Hospitals, Ni: total number of Nurses in each selected Hospitals, ni: proportion of Nurses in each selected Hospitals, n: Total sample size.

For selecting study participants after proportional allocation of study subjects to each hospital, simple random sampling techniques were to be used (Sees Fig. 2 Schematic Presentation of the Sampling Procedure).

### Study variables

#### Dependent variables

Knowledge and Attitude towards COVID-19.

#### Independent variables

##### Socio-demographic and economic characteristics related variables

Age of participants, sex of participants, marital status, educational status, monthly income, working institution name.

##### Workplace related Variables

Work experience in a clinical area, Nurse's work per day, the overworked status of nurses, source of information on COVID-19.

##### Knowledge of nurses about COVID-19 related variables

Clinical Manifestation of Covid-19, source of infection of Covid-19, prevention methods of COVID-19, IP for COVID-19, transmission route of COVID-19 susceptibility to COVID-19, availability of COVID-19 vaccine, COVID-19 Antibiotics, isolation period of COVID-19, medical masks for COVID-19, the pathogenesis of COVID-19.

##### The attitude of Nurses about COVID-19-related factors

The attitude of the Black race towards COVID-19, Wearing material of COVID-19, Hand wash related to COVID-19, Clinical manifestation of COVID-19, Management of COVID-19, Condition of COVID-19, Control of COVID-19, Isolation of COVID-19, Medical staff participation in COVID-19, family members may get an infection by COVID-19.

### Operational definition

**Attitude:** Participants with score of greater than or equal to 6 attitude questionnaires answered where considered to have a favourable attitude, and those who scored less than 6 attitude questionnaires answered were considered to have an unfavourable attitude towards COVID-19 [8].

**Knowledge:** Appropriate responses from nurses about COVID-19 through the structured knowledge questionnaires answered with ≥8 correct responses (from 13 knowledge questions) were considered having good knowledge and those with <8 correct responses (from 13 knowledge questions) were considered having poor knowledge [8].

**A suspected case of COVID-19:** is a person presenting with fever (>38°C) or history of fever and symptoms of respiratory tract illness, e.g., cough, difficulty in breathing, and a history of travel to or residence in a country/area or territory reporting local transmission of COVID-19 disease during the 14 days prior to symptom onset [1].

**Probable case:** is a suspect case for which testing for COVID-19 is inconclusive [1].

**Confirmed case:** is a person with laboratory confirmation of COVID-19 infection, irrespective of clinical signs and symptoms [1].

### Data collection tools and techniques

#### Data collection tools

The Data was collected using a pretested and structured Self-administered questionnaire, which was adapted from WHO COVID-19 guidelines, Training manuals and published articles with some modifications to the local context [[16], [17], [18], [19], [20], [21],^30^]). The questionnaires were prepared in the English version. The questionnaires addressed the provider and institutional characteristics of Nurses; the structured questions addressed the knowledge of Nurses towards COVID-19, and the Likert Scale guide addressed the Attitude of nurses towards COVID-19.

#### Data collection techniques

A total of seven trained data collectors and three supervisors (who have experience in data collection done as a task force of COVID-19 and Quarantine Treatment Center of COVID-19) were selected. Two days of training was given for data collectors and supervisors regarding the study purpose, methodology, how to conduct and administer the self-administered questionnaire, how to take consent, keep confidentiality, and respect the right of the participants.

#### Data quality control

The quality of the data was assured by pretesting of questionnaire on 5% of the sample (9 Nurses) in Bahir Dar Felege Hiwot Hospital prior to the start of the actual study to test the fitness of the questionnaire for the study settings; based on the result of the pretest any ambiguous question will be modified for clarity and consistency. Training about the data collection tool, as well as data collection procedures (ways of approaching the eligible Nurses and how to obtain permission for Self-Administered Questionnaires) was given to data collectors and supervisors for a total of two days prior to the data collection process.

The objectives of the study were clearly explained to the data collectors as well as supervisors. The respondents were to give brief orientation before they are given the Questionnaires, and supervision will be done at the spot by the supervisors. Throughout the course of the data collection, Data collectors were supervised at each site, a regular meeting was held between the data collector's supervisor and the principal investigator to discuss the problem arising in each interview, and detailed feedback was provided to the data collectors.

In addition, the collected data were checked daily for completeness, accuracy, and clarity by supervisors. The reliability of the tool was determined based on the analysis result of the pretest (Cronbach's alpha). The principal investigator checked every questionnaire before data entry. The data were stored in the form of a file in a private secured place.

### Data processing and analysis

After checking the completeness of the data, it was entered into Epi info version 7.2.0.1, and then; it was export to SPSS Version 24 for analysis. Descriptive analysis was done by computing proportions and summary statistics. The association between each independent variable and the outcome variable was assessed by using binary logistic regression. All variables with P ≤ 0.2 in the bivariate analysis were included in the final model of multivariable analysis to control all possible confounders.

AOR along with 95% CI was computed and P-value < 0.05 was considered to declare factors that have a statistically significant association with the outcome by using multivariable analysis in the binary logistic regression. The goodness of fit was tested by the Hosmer-Lemeshow statistic test. Finally, the result is presented in the form of texts, tables, and graphs.

## Ethical consideration

Ethical clearance was obtained from the Ethical Review Committee of Debre Tabor University, Research and Community service Directorate. A letter of permission was given to the South Gondar zone health department and Debre Tabor town health office and each hospital. The patient data were assessed upon the approval of the medical director of each hospital. For ensuring confidentiality, the respondent identifier was not recorded in the data collection checklist, and the data were used only for the intended study.

## Results

### Sociodemographic and economic characteristics of the study population

**Fig. 3:**
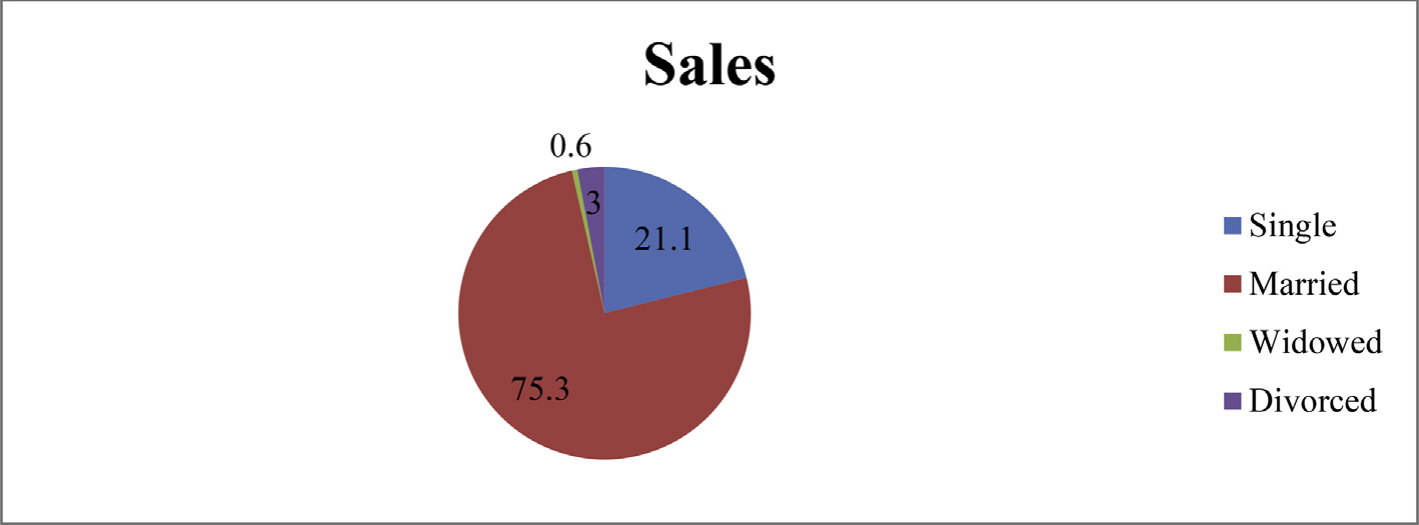
Distribution of marital status of study participants in in South Gondar Zone, Hospitals, Northwest Ethiopia (N = 166).

According to the study, 95 (57.2%) of the participants were females, and 71 (42.8%) were males. The largest proportion, 69 (41.6 %) of the respondents, was between the ages of 20 and 29 years, and the smallest proportion 29 (17.5 %) was ≥40 years. The majority, 121 (72.9%) of the respondents, were first degree holders, and 45 (27.1%) were diploma holders. Out of the total participants, 125 (75.3%), 35 (21.1%), 5(3%), 1(0.6%) were married, single, divorced, and widowed, respectively. Regarding monthly income, 100 (60.2%) had ≥5000 monthly income, 63 (38.0%) had 3001–4999 monthly income, 3 (1.8%) had <3000 monthly income (Table 1, Fig. 3).

**Table 1:** Socio-demographic and Economic characteristics of study participants in South Gondar Zone, Hospitals, Northwest Ethiopia (N = 166)

Variable	Category	Frequency	Percent (%)
Name of Hospitals where Nurses work	Debre Tabor General Hospital	75	45.2%
	Ebinat District Hospital	9	5.4%
	Adiss Zemen District Hospital	20	12%
	Nifas Mewucha District Hospital	13	7.8%
	Tach Gayint District Hospital	14	8.4%
	Simada District Hospital	13	7.8%
	Mekane Eyesus District Hospital	13	7.8%
	Andabet District Hospital	9	5.4%
Sex	Male	71	42.8%
	Female	95	57.2%
Age	20–29	69	41.6%
	30–39	68	41%
	≥40	29	17.5
Marital Status	Single	35	21.1%
	Married	125	75.3%
	Widowed	1	0.6%
	Divorced	5	3%
Educational Status	Diploma	45	27.1%
	1st Degree	121	72.9%
Monthly Income	<3000	3	1.8%
	30,001–4999	63	38%
	≥5000	100	60.2

### Workplace related characteristics of the study population

Among study participants, 64 (38.6%), 62 (37.3%), and 40 (24.1%) of the study participants had work experience, in the clinical area, of 5–10 years, less than 5 years, and greater than 10 years respectively. The majority, 142 (85.5%) of the study participants, had overworked status per day. In the source of information on COVID-19, the majority, 103 (62%) of the participants headed from International health organizations, e.g., WHO (Table 2).

**Table 2:** Work place-related characteristics of study participants in South Gondar Zone, Hospitals, Northwest Ethiopia (N = 166)

Variable	Category	Frequency	Percent (%)
Work experience in clinical area	<5 years	62	37.3%
	5–10 years	64	38.6%
	≥10 years	40	24.1%
Nurses Overworked status per day	≤ 8 hours	24	14.5%
	>8 hours	142	85.5%
Source of information on COVID19	International health organization e.g., WHO	103	62%
	Government sites and media e.g., MOH of Ethiopia	86	51.8%
	Social media e.g., WhatsApp, Facebook	75	45.2%
	News media e.g., TV, radio, newspaper	64	38.6%
	Journals	18	10.8%
	Others	1	0.6%

### Knowledge about COVID-19 characteristics of the study population

According to the study, 146 (88.0 %) respondents knew the symptoms of COVID-19; the majority, 122 (73.5%) of participants knew that the main symptoms of COVID-19 were fever and dry cough. Among the participants, 138 (83.1%) study participants, knew the source of infection of COVID-19, and 142 (85.5%) knew the prevention methods of COVID-19. The majority, 152 (91.6%) of participants, knew the period of incubation for COVID-19, among which 123 (74.1%) knew that the response period of incubation for COVID-19 was 1∼ 14 days. The majority, 133 (80.1%) of the study participant's responses was that the type of infectious disease for COVID-19 was viral. Regarding the Transmission route of COVID-19, which the majority of 165 (99.4%) knew, 134 (80.7%) of them knew it to be respiratory droplets and closeness. In susceptibility to COVID-19, 96 (57.8%) people's responses are generally susceptible to the overall knowledge of nurses towards COVID-19. There is currently no effective cure for COVID-2019, but early symptomatic and supportive treatment can help most patients recover from the infection; not all persons with COVID-2019 will develop into severe cases. Only those who are elderly, have chronic illnesses, and are obese, are more likely to be severe cases. It is not necessary for children and young adults to take measures to prevent the infection by the COVID-19 virus; people who have contact with someone infected with the COVID-19 virus should be immediately isolated in a proper place. In general, the observation period is 14 days; eating or contacting wild animals would result in infection by the COVID-19 virus. Persons with COVID-2019 cannot transmit the virus to others when a fever is not present. The COVID-19 virus spreads via the respiratory droplets of infected individuals. Wearing general medical masks can prevent one from acquiring infection by the COVID-19 virus. To prevent the infection by COVID-19, individuals should avoid going to crowded places such as bus terminals and avoid taking public transportation. Isolation and treatment of people who are infected with the COVID-19 virus are effective ways to reduce the spread of the virus. The isolation period is 2 weeks for COVID-19. COVID-19 vaccine is available in the market. Antibiotics are the first-line treatment for COVID-19. The knowledge on questions was calculated based on < 16 by giving 0 for non-correct answers and 1 for correct answers for all the 13 knowledge questions. Among respondents, 84.9% of nurses had good knowledge, and the rest had poor knowledge on COVID-19 (Table 3).

**Table 3:** Knowledge-related characteristics of study participants in study area (N = 166)

Variable	Category	Frequency	Percent (%)
Knew symptoms of COVID-19	yes	146	88.0 %
	no	20	12.0%
Knew main symptoms of COVID-19	Fever and dry cough.	122	73.5%
	Fatigue	4	2.4%
	Stuffy and runny nose	15	9.0%
	Sore throat and myalgia.	15	9.0%
	Diarrhea	4	2.4%
	I don’ know.	1	0.6%
Knew source of infection COVID-19	yes	138	83.1 %
	no	28	16.9%
a source of infection COVID-19	Anyone residing in or travelled to affected areas, contacts/travelling with someone having symptoms of severe acute respiratory infection.	89	53.6%
	the air by coughing and sneezing, close personal contacts, such as touching and shaking hands, touching contaminated objects or surfaces,	90	54.2%
	touching mouth, nose, eyes before washing hands	41	24.7%
	rarely faecal contamination	28	16.9%
prevention methods of COVID-19	yes	142	85.5 %
	no	24	14.5%
a prevention method of COVID-19	Maintaining basic hand and respiratory hygiene (include regular hand washing, covering mouth and nose when coughing and sneezing)	113	68.1%
	Safe food practices, thoroughly cooking meat and eggs	53	31.9%
	Avoiding close contact with anyone showing symptoms of respiratory illness such as coughing and sneezing	65	39.2%
	Avoiding close contact with live or dead farm or wild animals	51	30.7%
Knew period of incubation for COVID-19	yes	152	91.6 %
	no	14	8.4%
Knew period of incubation for COVID-19	1∼ 14 days	123	74.1%
	3 ∼ 7 days.	5	3.0%
	More than 14 days	21	12.7%
	I don’t know	1	0.6%
Knew Type of infectious Disease is COVID-19	Bacterial	28	16.9%
	Viral	133	80.1%
	I don’t know.	5	3.0%
Knew Transmission route of COVID-19	yes	165	99.4%
	no	1	0.6%
The main transmission route of COVID-19	Respiratory droplets and close	134	80.7%
	Water.	20	12.0%
	Food.	5	3.0%
	I don’t know	4	2.4%
Susceptible to COVID-19	The old and children	41	24.7%
	People are generally susceptible	96	57.8%
	Young adults	4	2.4%
	People with pre-existing diseases	22	13.3%
	I don’t know.	3	1.8%
There is currently no effective cure for COVID-2019, but early symptomatic and supportive treatment can help most Patients recover from the infection	True	128	77.1%
	False	38	22.9%
Not all persons with COVID-2019 will develop to severe cases. Only those who are elderly, have chronic illnesses, and are Obese are more likely to be severe cases.	True	114	68.7%
	False	30	30.7%
	I don’t know.	1	0.6%
It is not necessary for children and young adults to take measures to prevent the infection by the COVID-19 virus.	True	82	49.4%
	False	82	49.4%
	I don’t know.	2	1.2%
People who have contact with someone infected with the COVID-19 virus should be immediately isolated in a proper Place. In general, the observation period is 14 days.	True	132	79.5%
	False	34	20.5%
Eating or contacting wild animals would result in infection by the COVID-19 virus	True	111	66.9%
	False	46	27.7%
	I don’t know.	9	5.4%
Persons with COVID-2019 cannot transmit the virus to others when a fever is not present.	True	74	44.6%
	False	92	55.4%
The COVID-19 virus spreads via the respiratory droplets of infected individuals.	True	119	71.7%
	False	45	27.1%
	I don’t know.	2	1.2%
Wearing general medical masks can prevent one from acquiring infection by the COVID-19 virus.	True	102	61.4%
	False	64	38.6%
To prevent the infection by COVID-19, individuals should avoid going to crowded places such as bus parks and avoid taking public transportations	True	127	76.5%
	False	39	23.5%
Isolation and treatment of people who are infected with the COVID-19 virus are effective ways to reduce the spread of the virus.	True	125	75.3%
	False	38	22.9%
	I don’t know.	3	1.8%
The isolation period is 2 weeks for COVID-19?	Yes	125	75.3%
	no	41	24.7%
COVID-19 vaccine is available in markets	Yes	37	22.3%
	no	129	77.7%
Antibiotics are the first-line treatment for COVID-19?	Yes	40	24.1%
	no	126	75.9%

### Attitude about COVID-19 characteristics of the study population

Based on findings, 105 (63.3%) of respondents have a favourable attitude for COVID-19, whereas the rest 61 (36.7%) have an unfavourable attitude (Table 4).

**Table 4:** Attitude of nurses working in study area (N = 166)

Characters	S.Disagree		Disagree		Neutral		Agree		S. agree	
	No	%	No	%	No	%	No	%	No	%
Black race is protective towards COVID-19 disease.	103	62	30	18.1	3	3.6	21	12.7	3	3.6
Wearing a well-fitting face mask is effective in preventing COVID-19.	8	4.8	35	21.1	20	12.0	62	37.3	41	24.7
Using a hand wash can prevent you from getting COVID-19.	3	1.8	19	11.4	14	8.4	83	50.0	47	28.3
When a patient has signs and symptoms of COVID-19, I can confidently	1	0.6	29	17.5	34	20.5	75	45.2	27	16.3
Participate in the management of the patient.	6	3.6	14	8.4	32	19.3	75	45.2	39	23.5
Ethiopia is in a good position to contain COVID-19	14	8.4	28	16.9	26	15.7	70	42.2	28	16.9
COVID-19 will finally be successfully controlled?	46	27.7	16	9.6	27	16.3	40	24.1	37	22.3
Covid-19 patients should be kept in isolation	10	6.0	22	13.3	20	12.0	63	38.0	51	30.7
Medical staff are ready to participate in anti-epidemic in the community	4	2.4	8	4.8	28	16.9	74	44.6	52	31.3
You are worried one of your family members may get an infection	5	3.0	14	8.4	8	4.8	82	49.4	57	34.3

### Factors associated with the knowledge of nurses towards COVID-19

Variables having a p-value less than 0.2 during the bi-Variable analysis in the current study and variables considered as significant from other literature were analyzed; the multivariable analysis indicated that there was one variable that showed statistical significance with the Knowledge of Nurses towards COVID-19 in south Gondar Hospitals. Wearing general medical masks can prevent one from acquiring infection by the COVID-19 virus. AOR = 0.44, 95% CI = 0.005–0.362 were factors of knowledge about COVID-19 (Table 5).

**Table 5:** Factors associated with Knowledge of Nurses towards COVID-19 in study area, 2020

Variable	Category	Knowledge of nurses towards COVID-19		COR (95% CI)	AOR (95% CI)	pv
		Good	Poor			
There currently is no effective cure for COVID-2019, but early symptomatic and supportive treatment can help most Patients recover from the infection	True	104 (0.8)	24 (0.2)	.117 (.015-.896)		
	False	1 (0.03)	37 (0.97)	1		
It is not necessary for children and young adults to take measures to prevent the infection by the COVID-19 virus	True	62 (0.76)	20 (0.24)	.196 (.007–.552)		
	False	79 (0.94)	5 (0.06)	1		
Wearing general medical masks can prevent one from acquiring infection by theCOVID-19 virus.	True	78 (0.76)	24 (0.24)	0.052 (.0.07–0.392)	0.043 (0.005–0.362)	.004
	False	63 (0.98)	5 (0.02)	1	1	
COVID-19 vaccine is available in markets	no	117 (0.9)	12 (0.1)	5.28 (2.12–12.98)		
	yes	24 (0.65)	13 (0.35)	1		
Antibiotics are the first-line treatment for COVID-19?	no	117 (0.93)	9 (0.07)	8.66 (3.42–21.91)		
	yes	24 (0.6)	16 (0.4)	1		

### Factors associated with the attitude of nurses towards COVID-19

Variables having a p-value less than 0.2 during the bi Variable analysis in the current study and variables considered as significant from other literature were analyzed; the multivariable analysis indicated that there was one variable that showed a statistical significance with the the Attitude of Nurses towards COVID-19 in South Gondar Hospitals. Strongly agrees Medical staff are ready to participate in anti-epidemic in the community. (AOR = 0.08, 95% CI = (0.003–1.76) were factors of attitude about COVID-19 (Table 6).

**Table 6:** Factors associated with Attitude of Nurses towards COVID-19 in study area, 2020

Variable	Category	Attitude of nurses towards COVID-19		COR (95% CI)	AOR (95% CI)	PV
		Favourable	Un favourable			
Did you know prevention methods of COVID-19	yes	84 (0.6)	58 (0.4)	0.21 (0.59–0.72)		
	no	21 (0.88)	3 (0.12)	1		
What type of infectious Disease is COVID-19?	Viral	77 (0.58)	56 (0.42)	0.25 (0.09–0.68)		
	Bacterial	28 (0.85)	5 (0.15)	1		
There currently is no effective cure for COVID-2019, but early symptomatic and supportive treatment can help most Patients recover from the infection	True	73 (0.57)	55 (0.43)	0.052 (.0.07–0.392)		
	False	32 (0.84)	6 (0.16)	1		
Not all persons with COVID-2019 will develop to severe cases. Only those who are elderly, have chronic illnesses, and are Obese are more likely to be severe cases.	True	60 (0.63)	54 (0.47)	0.17 (0.07–0.47)		
	False	45 (0.87)	7 (0.13)	1		
Persons with COVID-2019 cannot transmit the virus to others when a fever is not Present.	false	54 (0.73)	20 (0.27)	0.46 (0.24–0.89)		
	true	51 (0.55)	41 (0.44)	1		
Wearing general medical masks can prevent one from acquiring infection by the COVID-19 virus.	true	57 (0.56)	45 (0.44)	0.42 (0.21–0.84)		
	false	48 (0.75)	16 (0.25)	1		
COVID-19 vaccine is available in markets	no	80 (0.62)	49 (0.38)	0.78 (0.36–1.70)		
	yes	25 (0.68)	12 (0.32)	1		
Antibiotics are the first-line treatment for COVID-19?	no	77 (0.61)	49 (0.39)	0.67 (0.31–1.45)		
	yes	28 (0.70)	12 (0.3)	1		
Medical staff are ready to participate in anti-epidemic in the community	S.agree	46 (0.88)	6 (0.12)	0.33 (0.27–4.19)	0.08 (0.003–1.76)	0.11
	agree	42 (0.57)	32 (0.43)	0.87 (0.11–7.05)	0.01 (0.00–0.54)	0.00
	Neutral	13 (0.46)	28 (0.54)	1.31 (0.18–9.83)	0.46 (0.01–0.21)	0.00
	D.agree	2 (0.25)	6 (0.75)	7.67 (0.91–64.9)	0.09 (0.28–0.31)	0.00
	SD. agree	2 (0.5)	2 (0.5)	1	1	

## Discussion

This study tried to address Knowledge, and Associated Factors towards COVID-19 among Nurses who work in South Gondar Zone Hospitals, Northwest Ethiopia 2020. The level of good Knowledge was found to be 84.9 % (95%CI: 77.8–90.4) among nurses towards COVID-19 in South Gondar Zone Hospitals. This result was higher than compared with other similar studies in Iran (56.5%). And to address Attitude, and Associated Factors towards COVID-19 among Nurses who work in South Gondar Zone Hospitals, Northwest Ethiopia 2020. The level of favourable attitude was found to be 63.3 % (95%CI: 54.3–72.1) among nurses towards COVID-19 in South Gondar Zone, Hospitals. This result was lower than compared with other similar studies in China (85%) feared self-infection with the virus; this level of knowledge finding was in line with a study conducted in Chinese health care workers (89%) [30], and in line with Japanese health care workers (89.5%) [31]. In line with a study conducted in Egypt, level of knowledge among attitude HCWs (80.4%) [32]. In a study done in Ethiopia, 88.2% of respondents had good knowledge [33]. The similarity of this study with China, Japan, Egypt, and Ethiopia could be the study design and educational professional resemblance among health care workers.

However, the level of knowledge result of this study was much lower than the study done in Pakistan (93.2%) [18]. The possible explanation for the difference could be study setting and individual health care provider's knowledge variability. The level of attitude results of this study was much lower than the study done in China (85%) [30], in Japan (77.7%), and Egypt 83.1% [31,32]. In a study done in Ethiopia, 94.7% of respondents had a positive attitude [33]. The possible justification for the difference could be studies setting, individual health care provider's knowledge variability, and training taken regarding COVID-19.

This level of knowledge result was higher as compared with other similar studies such as research done on Iranian nurses (56.5%) [16]. The possible justification for the difference could be the study period, which is the study conducted in Iran was around nearing the occurrence of COVID-19. In this study higher than with Study conducted in Amhara region, Ethiopia (70%), HWs had good knowledge of COVID-19 [34]. The possible justification for the difference could be the target population.

This level of Attitude result was higher as compared with other similar studies such as research done on Iranian health care professionals (21%) [20]. The possible justification for the difference could be the study period which is the study conducted in Uganda was around nearingthe occurrence of COVID-19.

In this study, in multivariable analysis, wearing general medical masks can prevent one from acquiring infection by COVID-19 virus has remained significantly associated with the level of knowledge dependent variable with 95% CI and P-value of <0.05; in multivariable analysis Medical staff are ready to participate in anti-epidemic in the community has remained significantly associated with the level of attitude dependent variable with at 95% CI and P-value of <0.05.

This study showed that wearing general medical masks can prevent one from acquiring infection by COVID-19 virus; AOR = 0.44, 95% CI = 0.005–0.362 were factors of knowledge about COVID-19. Whereas, ‘strongly agree’ Medical staff are ready to participate in anti-epidemic in the community: AOR = 0.08, 95% CI = (0.003–1.76 were factors of attitude about COVID-19.

This finding was not supported by the other studies because there is not enough study conducted.

## Limitation

It was based on online data collection techniques using email and telegram. Some health workers might not have access to such services due to limited access to technology, internet service and electric power. Thus, they might not be sampled even if they are important to this study. Also moreover, this study included nurses working only in government health facilities. Because it is a one-time study, it shared the limitations of a cross-sectional study to establish cause-effect relationships. The effect of improving the education of personnel and following up the improvement of practices.

## Conclusion

In this study, most of the nurses had good knowledge and a favourable attitude regarding COVID-19. Wearing general medical masks can prevent one from acquiring infection by COVID-19 virus were the factors in association with knowledge OF nurses on COVID-19. Similarly, Medical staff are ready to participate in anti-epidemic in the community factors associated with attitudes of nurses on COVID-19.

## Recommendations

### To health personnel/HEWs

❖ Shall prevent his/her self from COVID-19.

### To each hospital

❖ Providing Nurses, financial and administrative support is crucial.
❖ We recommend health education campaigns to the less educated nurses.
❖ Continuous provision of PPE and training of all nurses on proper infection prevention measures are serious and substantial.

### To the researchers

❖ It is better if a qualitative study is conducted.

## Abbreviation

AOR: Adjusted Odds Ratio
CDC: Communicable Diseases Control
COVID-19: Coronavirus 2019
CI: Confidence Interval
FMOH: Federal Ministry of Health
HCWs: Health Care Workers
IP: Incubation Period
KA: Knowledge, Attitude
KAP: Knowledge, Attitude, Practice
MOH: Ministry of Health
MOH: Ministry of Health
PCR: Polymerase Chain Reaction
SPSS: Statistical Package for Social Sciences
WHO: World Health Organization

## Availability of data and materials

Data will be available upon request from the corresponding author.
